# Deficiency of 25-Hydroxyvitamin D and Dyslipidemia in Indian Subjects

**DOI:** 10.1155/2013/623420

**Published:** 2013-12-18

**Authors:** Jaydip Ray Chaudhuri, K. Rukmini Mridula, Alluri Anamika, Demudu Babu Boddu, Pradeep Kumar Misra, A. Lingaiah, Banda Balaraju, Vcs Srinivasarao Bandaru

**Affiliations:** ^1^Department of Neurology, Yashoda Hospital, Hyderabad, India; ^2^Department of Neurology, Nizam's Institute of Medical Sciences, Hyderabad, India; ^3^Department of Biochemistry, Yashoda Hospital, Hyderabad, India; ^4^Department of Neurology, Caringhands Neurocenter, Vizag, India; ^5^Department of Medicine, Yashoda Hospital, Hyderabad, India; ^6^Director of Medical Services, Yashoda Hospital, Hyderabad, India; ^7^Department of Clinical Research, Yashoda Hospital, Hyderabad 500082, India

## Abstract

*Background*. Vitamin D deficiency is widespread throughout the world. Several reports have incriminated vitamin D deficiency as the cause of rickets, osteomalacia, and other chronic diseases. Recent studies have suggested a possible link between deficiency of 25-hydroxyvitamin D and dyslipidemia. *Aim*. To investigate the association between 25-hydroxyvitamin D deficiency and dyslipidemia in Indian subjects. *Methodology*. We recruited 150 asymptomatic consecutive subjects from patients' attendees at the Departments of Neurology and Medicine in Yashoda Hospital, Hyderabad, India. Study period was from October 2011 to March 2012. All subjects underwent 25-hydroxyvitamin D assay by chemiluminescent microparticle immunoassay, fasting blood sugar and lipid profile, calcium, phosphorus, alkaline phosphatase, and C-reactive protein (CRP). *Results*. Out of 150 subjects, men were 82 (54.6%), and mean age was 49.4 (±15.6) years. Among risk factors, hypertension was noted in 63/150 (42%), 25-hydroxyvitamin D deficiency in 59/150 (39.3%), diabetes in 45/150 (30%), dyslipidemia in 60 (40%), smoking in 35/150 (23.3%), and alcoholism in 27/150 (18%). Deficiency of 25-hydroxyvitamin D was significantly associated with dyslipidemia (*P* = 0.0001), mean serum glucose (*P* = 0.0002) mean CRP (*P* = 0.04), and mean alkaline phosphatase (*P* = 0.01). Multivariate analysis showed that 25-hydroxyvitamin D deficiency was independently associated with dyslipidemia (odds ratio: 1.9; 95% CI : 1.1–3.5). *Conclusions*. We found that deficiency of 25-hydroxyvitamin D was independently associated with dyslipidemia in Indian subjects.

## 1. Introduction

Vitamin D deficiency is a common disorder, found in all age groups and in both genders [1, 2]. It is prevalent in various parts of the world including India [1–3] with an increased occurrence in high and low latitude countries [4]. Worldwide, the prevalence of vitamin D deficiency is 50% in elderly [5] and within Europe in 2%–30% of adults [6]. 

Serum 25-hydroxyvitamin D level is a sensitive measure of vitamin D status of an individual [1], and the prevalence of inadequate 25-hydroxyvitamin D is around 30% to 50% in the general population [7]. 

Recent reports have found that hypo 25-hydroxyvitamin D is associated with atherosclerosis [8], obesity [9], diabetes [10], hypertension [11], myocardial infarction [12], and stroke [13]. Dyslipidemia is a an independent risk factor for cardiovascular and cerebrovascular diseases in individuals [14, 15]. Current studies have observed that low 25-hydroxyvitamin D is associated with dyslipidemia [16, 17]. We aim to investigate the relationship of circulating levels of 25-hydroxyvitamin D with dyslipidemia in Indian subjects. Very limited data were available. 

## 2. Material and Methods

One hundred and fifty asymptomatic subjects were consecutively enrolled from patients' attendees at the Departments of Neurology and Medicine, Yashoda Hospital Hyderabad, India, a major referral center in the south Indian state of Andhra Pradesh. The design of the study was approved by the Institutional Ethics Committee. The study period was from October 2011 to March 2012. Data were collected through face-to-face interviews of all subjects and medical record review by physicians. Standardized questions were adapted from the behavioral risk factor surveillance system [18], by the Centers for Disease Control and Prevention regarding the following conditions: hypertension, diabetes, hypercholesterolemia, peripheral vascular disease, cigarette smoking, and cardiac conditions such as myocardial infarction and coronary artery disease.

Subjects with prior history of cardiovascular diseases, cerebrovascular disorders, osteomalacia, any other bone diseases, bone pains, muscle weakness, and vitamin D deficiency or those who were on calcium, vitamin D supplements, and lipid lowering medications were excluded. Standard techniques were used to measure blood pressure, height, weight, and blood tests [19]. All subjects underwent fasting serum glucose, lipid panel (including total cholesterol, low density lipoprotein (LDL) cholesterol, high-density lipoprotein (HDL) cholesterol, very low density lipoprotein (VLDL) cholesterol, and triglycerides), calcium, alkaline phosphatase, phosphorus, and C-reactive protein (CRP). In lipid panel, total cholesterol, VLDL cholesterol, HDL cholesterol, and triglyceride levels were measured directly. If plasma triglyceride levels were less than 300 mg/dL, LDL cholesterol levels were calculated by the Friedewald formula. If plasma triglycerides were more than 300 mg/dL, direct assay of LDL cholesterol was done.

### 2.1. Risk Factor Assessment

Hypertension was defined (Joint National Committee VII) as a systolic blood pressure >140 mm Hg and/or a diastolic blood pressure >90 mm Hg based on the average of 2 blood pressure measurements or a patient's self-reported history of hypertension or antihypertensive use [20]. Patients with fasting plasma sugar more than 110 mg/dL were considered as diabetic [21]. Dyslipidemia was defined (ATP III) as one or more of the following: total cholesterol more than 200 mg/dL, low density lipoprotein-cholesterol (LDL-C) more than 130 mg/dL, high-density lipoprotein-cholesterol (HDL-C) below 40 mg/dL, very low density lipoprotein-cholesterol (VLDL-C) more than 30 mg/dL, and triglycerides more than 150 mg/dL [22]. Alcoholics were defined as those in whom the alcohol consumption was >50 g/day (equivalent to 500 mL [2 drinks] of wine, 1000 mL of beer, or more than 5 drinks [units] of spirits) [23]. Body mass index (BMI) values more than 30 kg/m^2^ were considered as obese [24]. Smokers were defined as those reporting daily smoking. Exsmokers and occasional smokers were classified as nonsmokers [25].

### 2.2. Estimation of 25-Hydroxyvitamin D

We used chemiluminescent microparticle immunoassay (CMIA) with automated instrument for estimation of 25-hydroxyvitamin D. This instrument's sensitivity and specificity were 53% and 90.5%. As per current literature [1] and according to lab manual, we considered values ≤20 ng/mL as 25-hydroxyvitamin D deficiency and more than 20 ng/mL as normal.

### 2.3. Statistical Analysis

Statistical analysis was performed using SPSS 16.0 window software (statistical package for the Social sciences, SPSS Inc.). Continuous variables were presented in titer of mean + SD. Categorical variables were expressed as proportions. The Student's *t*-test was performed to test the differences in continuous variables, and *χ* ^2^ test was used to study the association in proportions. All tests were two sided and *P* value <0.05 was considered statistically significant. Pearson's product moment correlation coefficient “*r*” was used to evaluate the strength of the association between 25-hydroxyvitamin D levels and lipid panel as well as CRP levels. Multiple logistic regression was performed for all risk factors, age, gender, hypertension, diabetes, smoking, alcoholism, dyslipidemia, and obesity.

## 3. Results

In our study, amongst 150 subjects, the number of men was 82 (54.6%) and mean age was 49.4 ± 5.6 yrs. Hypertension was noted in 63 subjects (42%), deficiency of vitamin D3 in 59 subjects (39.3%), followed by dyslipidemia in 60 subjects (40%), and diabetes in 45 (30%) subjects, and 35 (23.3%) subjects were smokers (Table 1). Of the 59 (39.3%) subjects with 25-hydroxyvitamin D deficiency, men were 32, mean age was 50.1 ± 15.1 years with an age range of 25–82 years. On comparison between subjects with normal versus deficiency 25-hydroxyvitamin D levels, significant association was seen with dyslipidemia (*P* = 0.02) and elevated CRP (*P* = 0.004) in 25-hydroxyvitamin D deficiency group. Significant association with higher mean serum glucose levels and mean CRP levels was noted in 25 hydroxy vitamin D deficiency group (Table 2).

**Figure 1:**
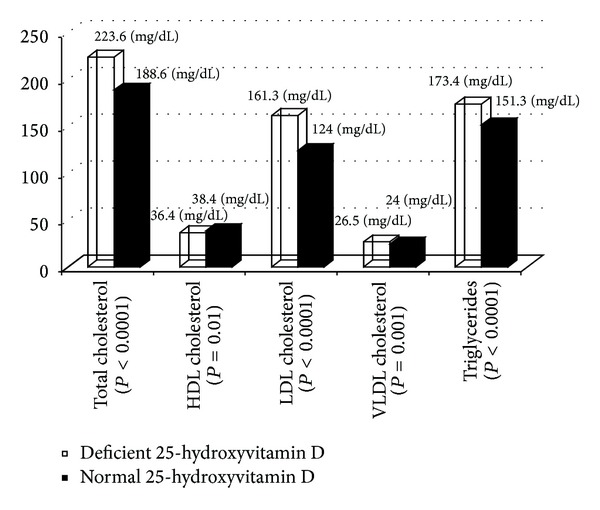
Comparison between 25-hydroxyvitamin D normal subjects and 25-hydroxyvitamin D deficient subjects with lipid profiles (mean levels).

Elevated mean total cholesterol, LDL cholesterol, VLDL cholesterol, triglyceride levels, and low HDL-C levels were significantly associated with 25-hydroxyvitamin D deficiency, when compared to subjects with normal 25-hydroxyvitamin D levels (Figure 1). We evaluated the strength of the association of 25 hydroxyvitamin D levels and the lipid panel. There was weak but significant (*P* < 0.05) inverse correlation between 25 hydroxyvitamin D levels and total cholesterol (*r* − 0.25), LDL cholesterol (*r* − 0.3), triglyceride levels (*r* − 0.2), and CRP levels (*r* − 0.3). A positive correlation was noted with HDL cholesterol level (*r*  0.37).

The combination of the associated markers that is, elevated CRP with dyslipidemia, had a more robust association with 25-hydroxyvitamin D deficiency compared to normal 25-hydroxyvitamin D groups (*P* = 0.002) (Table 3). The association of 25-hydroxyvitamin D deficiency with dyslipidemia persisted even after multiple logistic regression analysis showed independent association (odds ratio of 1.9 : 95% CI : 1.1–3.5) (Table 4).

## 4. Discussion

Our study demonstrated deficiency of 25-hydroxyvitamin D in 39.1% of asymptomatic Indian subjects and conforms to previous reports [26, 27]. The increasing prevalence of vitamin D deficiency inspite of available sunshine is multifactorial. The high fiber diet with phosphates and phytates which deplete vitamin D leads to reduced oral intake. As most of our populations are dark in complexion, the increased melanin may reduce vitamin D production by absorbing UVB rays [28]. Other factors like increased indoor occupational activities, increased pollution, and genetic predisposition contributes to vitamin D deficiency [29].

### 4.1. 25-Hydroxyvitamin D and Gender

The present study showed an equal distribution of deficiency of 25-hydroxyvitamin D in both genders, men 32 (54.2%) and women 27 (45.8%). Similar reports with equal distribution amongst both sexes were observed from Pakistan. [30]. However, some reports have established more prevalence in women than in men [31]. The most likely reason may be due to less exposure to sun in some regions of the world and reduced intake. This has been noted to cause increased bone turnover after the age of 40 in women. All biochemical markers of bone turnover tend to increase at and after menopause with a decline in serum estrogen levels. In contrast, in men, all biochemical markers of bone turnover except serum total alkaline phosphatase have a tendency to decrease with age [32].

### 4.2. 25-Hydroxyvitamin D and Serum Calcium, Phosphorus, and Alkaline Phosphatase

Vitamin D is a key monitor of calcium metabolism and is required for sufficient absorption of calcium from diet [7]. Deficiency of vitamin D leads to increased expression of parathormone and release of calcium from bone. This activity is reflected by increased alkaline phosphatase. The mean alkaline phosphatase was significantly increased (120 ± 8.8) with low 25-hydroxyvitamin D levels, suggesting increased bone turnover, similar to the existing literature [6, 33].

### 4.3. 25-Hydroxyvitamin D and Atherogenic Risk Factors

In the present study, 42% of our subjects had hypertension, 30% had diabetes, 23.3% were smokers, and alcoholism was seen in 18% while obesity was noted in 1.8%. We observed no significant relationship between low 25-hydroxyvitamin D levels and subjects with hypertension, smoking, and alcoholism. We found that mean serum glucose level was significantly higher in subjects with low 25-hydroxyvitamin D. Cigolini et al. showed that subjects with low 25-hydroxyvitamin D were more significantly associated with type 2 diabetes compared to controls (60.8% versus 42.8%) as well as higher levels of HbA1c [34]. Previous studies have also found association of hypo 25-hydroxyvitamin D with impaired fasting glucose, risk of type 2 diabetes mellitus, and hypertension [11, 34, 35]. Our study may be underpowered due to small numbers of subjects with these risk factors.

### 4.4. 25-Hydroxyvitamin D and Total Cholesterol

In the present study, we found significantly increased mean total cholesterol levels in asymptomatic Indian subjects with deficiency of 25-hydroxyvitamin D compared to those with normal 25-hydroxyvitamin D subjects. Karhapää et al., in his study, found that hypovitaminosis D was associated with high total cholesterol in Belgian men [16]. Auwerx et al. observed increased total cholesterol levels associated with deficiency of 25-hydroxyvitamin D in Finnish people [17]. Martins et al. studied non-Hispanic black and Mexican American individuals of the age of 60 and older and noted elevated total cholesterol levels with hypo 25-hydroxyvitamin D [36]. Similar findings were detected in Korean adults [37].

### 4.5. 25-Hydroxyvitamin D and HDL Cholesterol

In our study, we found significantly lower levels of high-density lipoprotein in subjects with deficiency of 25-hydroxyvitamin D, which was similar to other studies [38, 39]. Auwerx et al. from Belgium showed a significant association of high HDL cholesterol with normal 25-hydroxyvitamin D in both genders [16]. Choi et al. [37] from Korea observed that low HDL cholesterol was associated with hypo 25-hydroxyvitamin D. This association of low vitamin D with the decrease in HDL cholesterol seems to start very early in life as has been reported in Russian [40] and Spanish children [41].

### 4.6. 25-Hydroxyvitamin D and LDL Cholesterol

In the present study, <20 ng/mL of 25-hydroxyvitamin D was significantly associated with elevated LDL cholesterol and this reiterates the findings of Auwerx et al. from Finland [17]. The mechanism of association of hypovitaminosis D with cholesterol in human is not clearly known. It may be via photometabolism. In the presence of sunlight, squalene in exposed skin is converted into 7-dehydrocholesterol and vitamin D (and photometabolites of vitamin D); in the absence of effective sunlight, its metabolic pathway is diverted into the formation of cholesterol [35].

In addition, the 7-dehydrocholesterol pathway in the liver is common to cholesterol and 25-hydroxyvitamin D synthesis [42]. Further, the levels of 1,25 hydroxyvitamin D have been observed to have “extrahepatic” links with HDL cholesterol and ApoA1. In hypothesis, it is likely that metabolism of cholesterol and insulin sensitivity are influenced by 1,25-hydroxyvitamin D at various levels within and outside liver.

### 4.7. 25-Hydroxyvitamin D and Triglycerides

We also found a significant relationship between deficiency of 25-hydroxyvitamin D and high triglycerides. Cigolini et al. from Italy [34], Martins et al. from USA [36], and Hyppönen et al. from UK [43] noted similar association. Botella-Carretero et al. showed increased triglycerides in women with obesity with hypo 25-hydroxyvitamin D compared to those with adequate 25-hydroxyvitamin [44]. Elevated triglycerides' association with low 25-hydroxyvitamin has been noted even in children [41]. 

### 4.8. Mechanism

Two main mechanisms have been postulated for vitamin D mediated reduction in serum triglycerides. First mechanism vitamin D increases serum calcium by enhancing intestinal calcium absorption. This calcium could then reduce serum triglycerides by reducing hepatic triglyceride formation and secretion [37]. Second mechanism is that vitamin D has a suppressive effect on serum PTH concentration. As plasma postheparin lipolytic activity is reduced by elevated PTH concentration [45], low serum PTH may reduce serum triglycerides via increased peripheral removal. Apart from the above, two other mechanisms have also been implicated. Vitamin D may regulate triglyceride metabolism by causing the expression of VLDL cholesterol receptors in some types of cell [46]. Another possible mechanism to explain the association between 25-hydroxyvitamin D and triglycerides would be through insulin resistance: when vitamin D deficiency is present, the risk of insulin resistance increases [37] and this is associated with an elevation of levels of VLDL cholesterol and triglycerides [47].

### 4.9. 25-Hydroxyvitamin D and CRP

We found the combination of positive CRP with dyslipidemia to be significantly associated with a deficiency of 25-hydroxyvitamin D. One of the recent study advocated the association of positive CRP with 25-hydroxyvitamin D [48]. Vitamin D acts as an anti-inflammatory agent by decreasing the production of proinflammatory cytokines and modulation of tissue specific immune response [49, 50]. The metabolic activities of vitamin D are mediated by its binding to high-affinity vitamin D receptor (VDR) which acts as a ligand-activated transcription factor. Deficiency of both vitamin D and VDR causes development of certain autoimmune diseases [51]. It has been suggested that vitamin D modulates the expression of several cytokine genes controlled by the VDR thus reducing inflammation [52]. Vitamin D reduces production of interleukin-2, interferons and stimulates the T-helper type 2 lymphocytes, which results in reduction of matrix metalloproteinases, restricting atherosclerotic plaque progression [50].

## 5. Conclusions

In this study, we established that low levels of 25-hydroxyvitamin D were independently associated with dyslipidemia. Hence 25-hydroxyvitamin vitamin D deficiency endangers this population for an early onset of cardiovascular and cerebrovascular diseases. Although this association is emerging clearly, studies on the effect of vitamin D supplementation on reducing dyslipidemia are contradictory and unclear. Further studies are required for confirming these findings.

## Figures and Tables

**Table 1:** Baseline characteristics.

Parameters	Numbers (*n* = 150)
Men	82 (54.6%)
Mean age	49.4 ± 15.6
Age range	19–82
Risk factors	
Hypertension	63 (42%)
Diabetes	45 (30%)
Smokers	35 (23.3%)
Dyslipidemia	60 (40%)
Alcoholics	27 (18%)
25-hydroxy vitamin D deficiency	59 (39.3%)
Obesity	2 (1.3%)
C-reactive protein	71 (47.3%)

**Table 2:** Comparison between 25-hydroxyvitamin D normal subjects and 25-hydroxyvitamin D deficient subjects.

Parameters	25-Hydroxyvitamin D normal subjects (*n* = 91)	25-Hydroxyvitamin D deficient subjects (*n* = 59)	*P* value
Men	48 (52.7%)	32 (54.2%)	0.7
Women	43 (47.3%)	27 (45.8%)	0.8
Age range	19–76	25–82	
Mean age	49 ± 16.1	50.1 ± 15.1	0.6
Hypertension	35 (38.4%)	28 (47.4%)	0.3
Mean fasting glucose (mg/dL)	131.8 ± 13.5	149.9 ± 35.2	0.0002
Obesity	2 (2.7%)	0 (0)	0.4
Smoker	20 (21.9%)	15 (25.4%)	0.2
Alcoholics	15 (16.4%)	12 (20.3%)	0.3
Mean CRP (mg/dL)	0.5 ± 0.4	0.8 ± 0.4	0.04
Median CRP (mg/dL)	0.4	0.8	0.04
Dyslipidemia	28 (30.7%)	32 (54.2%)	=0.0001
Mean alkaline phosphatase	96.8 ± 5.6	120 ± 6.7	=0.01
Mean serum calcium	8.9 ± 1.7	7.9 ± 1.8	0.7
Mean serum phosphorus	2.6 ± 0.7	2.4 ± 0.9	0.8

**Table 3:** Association of combined C-reactive protein and dyslipidemia, with 25-hydroxy vitamin D levels.

	25-Hydroxyvitamin D normal subjects	25-Hydroxyvitamin D deficient subjects	*P* value
Positive CRP + dyslipidemia (*n* = 35)	12 (34.2%)	23 (65.6%)	=0.002
Positive CRP + normal lipids (*n* = 36)	22 (61.1%)	14 (38.8%)	=0.01

**Table 4:** Odds ratio analysis, before and after adjustment, between deficiency of 25-hydroxyvitamin D and various risk factors.

	Univariate analysis		Multivariate analysis	
Risk factors	Odds ratio	95% CI	Odds ratio	95% CI
Hypertension	1.4	0.7–2.8	0.9	0.4–1.4
Diabetes	1.3	0.6–2.7	0.7	0.4–1.1
Dyslipidemia	2.6	1.3–5.2	1.9	1.1–3.5
Smoking	1.2	0.5–2.6	∗	∗
Alcoholism	1.2	0.5–3.0	∗	∗

*Number of patients insufficient for statistical analysis.
